# The Impact of Re-tear on the Clinical Outcome after Rotator Cuff Repair Using Open or Arthroscopic Techniques – A Systematic Review

**DOI:** 10.2174/1874325001711010095

**Published:** 2017-02-28

**Authors:** Ilias Galanopoulos, Aslanidis Ilias, Konstantinos Karliaftis, Dimitrios Papadopoulos, Neil Ashwood

**Affiliations:** 1 Department of Orthopedics, 401 General Military Hospital of Athens, Athens, Greece; 2 General Hospital of Athens “Korgialeneio-Benakeio”- Hellenic Red Cross, Athens, Greece; 3 Department of Orthopedics, Queen’s Hospital, Burton-on-Trent, United Kingdom

**Keywords:** Double-row repair, Failed rotator cuff repair, Single-row repair, Structural failure, Tendon healing

## Abstract

**Background:**

It is generally accepted that rotator cuff repair gives satisfactory results in the long term, although most studies have so far shown a fairly high rate of structural failure or re-tear. The purpose of this review study is to assess whether failure of the repaired cuff to heal could negatively affect the functional outcome.

**Methods:**

This article includes an extensive Internet PubMed based research in the current English-language literature including level I to level V studies as well as systematic reviews.

**Results:**

According to this extended study research, the results are mixed; certain reports show that patients with a healed rotator cuff repair have improved function and strength compared to those with structural failure, whereas other studies support the generally perceived concept that tendon re-tear does not lead to inferior clinical outcome.

**Conclusion:**

Further high-level prospective studies with larger numbers of patients and longer follow up are needed to overcome the current debate over function between healed and failed rotator cuff repairs.

## INTRODUCTION

Rotator cuff repair can reliably improve shoulder function and decrease pain with both open and arthroscopic techniques. Several biomechanical studies have demonstrated that double-row transosseous equivalent repair techniques result in stronger initial fixation of tendon to bone, which may lead to improved healing of the rotator cuff [1]. Interestingly, despite the evolution of repair techniques and the development of instrumentation and suture anchors, the rate of unhealed or recurrent rotator cuff tears remains relatively high (in many studies >20%). Furthermore, previous studies attempting to correlate patient outcome to structural integrity of the rotator cuff repair have not demonstrated definitive results [2, 3].

It is universally accepted that most patients treated with rotator cuff repair do well regardless of the structural integrity of the repair. There are studies in the literature suggesting that patients with a re-rupture after rotator cuff repair still have significant improvement compared with their preoperative state [4-7]. The re-rupture usually is smaller than the original tear, and the structural failures are tolerated well, with satisfactory pain relief and functional improvement, including abduction strength. However, several other reports have shown a direct correlation between the postoperative clinical outcome and anatomic healing of the rotator cuff [8-13]. There is still lack of high-level double blinded prospective studies that directly assess the impact of rotator cuff re-tear on the functional outcome. The aim of our study is to review the highest-level studies available that report both the structural integrity of the repaired rotator cuff and the patient’s clinical outcome. The hypothesis was that failed rotator cuff repair would result in suboptimal clinical outcome compared with structurally healed repair.

## FACTORS PREDICTING ROTATORS CUFF RE-TEAR RATE

There are several factors that seem to increase the risk of rotator cuff re-tear after surgical repair, open or arthroscopically. The difficulty for researchers is to assess the statistical significance of each of them separately. Older age, larger tear size, thickness of tear, greater muscle-tendon unit retraction and poor muscle quality have all seemed to negatively affect tendon healing [14, 15]. With regards to the tear size, both the anteroposterior and mediolateral tear length also seem to affect the incidence of recurrence [16]. Tear size areas <2cm^2^ have higher healing rates and successful rotator cuff integrity maintenance than those with >6cm^2^ [17]. We should also mention that there are published studies that compared the outcomes after arthroscopic repair of partial versus small or medium-sized full-thickness rotator cuff tears [1, 18], which have shown that the clinical outcome and retear rate after repair of partial-thickness rotator cuff tears are similar to those after repair of full-thickness tears.

Numerous studies have shown that fatty degeneration [19-21] of the rotator cuff muscle negatively affects tendon healing. Most of them show a statistically significant correlation between the level of fatty infiltration and the rate of re-rupture in the long-term period [21, 22]. Especially the presence of atrophy at the infraspinatus and the reduced acromiohumeral distance seem to be the most important parameters [23]. In addition, the intensity and type of daily activities could play a significant role; thus high re-tear rates have been observed in heavy worker groups [24, 25]. Revision surgery has probably higher possibility of failure than primary repair and is associated with increased pain, impaired overhead function, less passive motion, diminished strength, and less overall satisfaction with poorer overall shoulder function [26].

Smoking, osteoporosis, diabetes and hypercholesterolemia can all negatively affect tendon healing [14]. The presence of diabetes does not seem to affect range of motion, pain and function of the shoulder. However, sustained hyperglycemia increases the possibility of anatomic failure at the repaired site, whereas an effective glycemic control is probably associated with better overall results [27].

The duration of shoulder immobilization [28] is also a significant predictor factor for structural failure. The results of various studies showed lower re-tear rates and better clinical scores in patients treated with immobilization for 8 weeks compared to those treated with immobilization for 4 weeks. Meta-analysis of 37 studies [29] not only approved this correlation but also found that in both small and large rotator cuff tears early active range-of-motion (ROM) was associated with increased risk for post-operative failure compared to late onset of active ROM exercises. Nevertheless, there are some level I and level II randomized control trials comparing prolonged immobilization with early initiation of passive motion exercises that found no significant difference in healing rates, ASES, SST, Constant and VAS scores postoperatively [30].

## DOES THE TECHNIQUE AFFECT ROTATOR CUFF HEALING RATE? (Table **1**)

More recently, investigators have attempted to correlate the integrity of the arthroscopic repair with postoperative function and have demonstrated widely varying results, with generally high failure rates [20, 31]. This observation has led to the design of a number of studies in order to analyze various repair techniques and to compare open and arthroscopic repair including both single-row and double-row techniques.

Double-row repairs seem to be biomechanically stronger compared with transosseous or single-row repairs at least for all tears greater than 1 cm [32]. However, there are meta-analyses which claim that double-row technique did not lead to a statistically significant improvement in clinical performance or radiographic healing after a long-term follow up [33]. In addition a few level I and level II studies [34] which compared functional outcome and structural integrity between these two techniques showed that single-row repairs achieved similar clinical outcomes to those after double-row repairs, although there was a trend toward a lower re-tear rate with the double-row technique. Biomechanically this seems to be important only for specific groups of patients such as athletes, young people and heavy workers, who want to maintain the muscle strength of the rotator cuff to a similar level to that before the tear [17]. At this point it is important to notice that double row techniques with excessive tension may lead to rupture at the muscolotendinous junction [5, 15, 35, 36]. According to several studies, suture-bridge technique leads to better functional outcomes, lower rates of re-tear and higher patient satisfaction compared with the traditional double-row technique in full-thickness rotator cuff tears [37, 38].

Use of platelet rich plasma is another adjunctive technique which is used along with tendon repair particularly in massive tears and revision cases, but unfortunately there is still lack of robust evidence to support the wide use of it. Further research is needed to identify effective biologically directed augmentations that will improve structural healing [39]. In this effort to improve the biological environment at the surgical site, studies have been conducted to assess the significance of multiple channeling in the greater tuberosity in an effort to achieve enhanced healing by the presence of mesenchymal stem cells [40, 41]. Postoperative results showed that although the re-tear rate was significantly lower in the groups with the addition of multiple channels, there is no significant difference in clinical outcomes for the patients.

Finally, different healing tissue has been observed after the various techniques used. There are a lot of studies that claim differences, according to the technique, to the expression of type I and III collagen in the tendon-to-bone junction that affects healing process and re-tear rate [1, 42]. It seems that type III collagen was detectable for longer time postoperatively in single-row patients group than in double-row patients group. An important observation is that the increase in the expression of type II collagen and clusters of chondrocytes were observed only in the double-row group after the operation.

Recent studies that analyze whether or not there is statistically significant difference between repair techniques of rotator cuff tears are included in Table **1**.

## CLINICAL STUDIES SHOWING BETTER RESULTS IN PATIENTS WITH HEALED REPAIR (Table **2**)

Recurrent or persistent defects after rotator cuff repair (RCR) are common. Retears have been documented in 13% to 57% of patients after open repair [43, 44]. Goutallier *et al*. [21] stated that if the fatty degeneration index is 2 or less, open tension-free tendon-to-bone suture repair is effective functionally and structurally, if the repair remains intact after 1 year. After repair of tears smaller than 3 cm, both open and arthroscopic RCR provided reliably satisfactory clinical results, with a high rate of cuff integrity evident after both types of repair at a minimum of 1 year postoperatively. In tears larger than 3 cm, cuff integrity was greater after open than arthroscopic repair. Many authors have found that chronic and massive rotator cuff tears have a high likelihood for re-tear after either open or arthroscopic technique used [6, 16, 23, 24, 45].

In a level IV study, Vastamaki *et al*. [46] studied long-term cuff integrity after open rotator cuff repair and tried to determine whether their findings correlated with clinical and functional results. They retrospectively evaluated 67 patients using MR arthrography with a minimum follow-up of 16 years. Their results showed a re-tear rate of 94% with concomitant fatty infiltration and a direct correlation between clinical results and cuff integrity: patients with an intact rotator cuff or a small re-tear (< 4cm^2^) had greater strength than patients with larger re-tears.

Park **et al*.* [47], in a retrospective level IV study including 36 patients with massive rotator cuff tears, evaluated the clinical and ultrasonographic outcomes of arthroscopic suture bridge repair. Their findings showed a 25% failure rate with larger re-tears leading to poorer functional outcomes compared with patients with smaller ones. Kim *et al*. [48], in a level III case control study including 66 patients, evaluated clinical outcomes and MRI findings after arthroscopic suture bridge repair of massive rotator cuff tears. Their results showed a 42.4% re-rapture rate at a mean follow-up of 25.4 months with clinical scores in the completely healed group being significantly better to those with failure recurrence (p<0.05). They also found that higher degree of fatty infiltration and greater degree of tendon retraction were the two most important negatively associated factors.

Zumstein *et al*. [45], in a long-term clinical study, evaluated clinical outcomes and structural integrity after open repair of rotator cuff tears (mean follow-up 9.9 years). They found a re-tear rate of 57%, with patients in the healed group achieving significantly better results than those with a failed reconstruction. They also noted that lateral extension of the acromion was a risk factor for recurrence.

Kim *et al*. [49] examined 180 patients performing ultrasonography to evaluate rotator cuff integrity and found that patient satisfaction, ASES and SST scores were significantly poorer in the re-tear group (p<0.05). Similarly to other studies the structural failure was approximately 26% but interestingly all three scores were significantly better in the oldest age category (p<0.05). Contrary to the generally perceived concept, their results imply that non-anatomic factors including younger age, lower education level, and a Workers' Compensation claim were associated with poorer outcomes.

Finally, Lafosse *et al*. evaluated a series of 105 patients undergoing arthroscopic double-row rotator cuff repair. The authors assessed the functional and anatomic results based on computed tomography or MRI arthrography in order to determine the postoperative tendon integrity [50]. The evaluation included determination of pain, strength, range of motion and Constant scores pre and postoperatively. In order to determine the impact of a failed repair on the clinical outcome, the authors directly compared the measured clinical parameters between patients with intact rotator cuff repair and those with structural failure. They concluded that the clinical outcome was superior in patients with healed repairs, although not statistically significant. Interestingly, pain was the only parameter in which a statistical significance was noted (p=0.014).

## CLINICAL STUDIES SHOWING NO DIFFERENCE IN CLINICAL OUTCOME BETWEEN PATENTS WITH HEALED AND STRUCTURALLY FAILED ROTATOR CUFF (RC) REPAIRS (Table **3**)

Jost *et al*. [44] in a prospective study tried to evaluate the clinical outcomes of a consecutive series of rotator cuff re-ruptures after repair. They concluded that patients with a re-rupture after rotator cuff repair still had significant improvement compared with the preoperative state. The post-operative defect usually was smaller than the original tear, and the structural failures were tolerated well, with good pain relief and functional improvement, including abduction strength. These findings suggest that the potential for structural failure should not be considered to be a formal contraindication to an attempt at rotator cuff repair if optimal functional recovery is the goal of treatment.

Voigt *et al*. [35], in a retrospective level IV clinical study including 51 patients who had undergone an arthroscopic suture bridge repair of supraspinatus tear, evaluated structural integrity by MRI scan 12 months postoperatively and assessed clinical improvement by SST and Constant scores. Their results showed a re-tear rate of 28.9% with no significant difference in the clinical outcome between the intact and non-intact repairs suggesting that structural failure is not identical to clinical failure. They also noted that patient age more than 60 was found to negatively influence tendon healing. Similarly, Kim *et al*. [37], in a series of 77 patients who underwent arthroscopic suture bridge repair of full thickness cuff tears, came to the conclusion that postoperative clinical outcomes improved in all patients and did not differ significantly between patients with healed rotator cuff and those with structural failure (p = 0.438, p = 0.625, and p = 0.898 for the UCLA, ASES, and Constant-Murley scores, respectively).

Larger case series [51] compared clinical and structural outcomes of rotator cuff repairs in 238 patients younger and older than 70 years. They concluded that both groups showed significant improvements in clinical outcomes with no significant difference between the two populations, despite the high RC failure rate (39.8% in the younger group, 51.1% in the older group confirmed by MRI scan at least six months postoperatively (p=0.161)). Interestingly the authors found negative influence of the intraoperative tear size but not of the increased age.

Sugaya *et al*. [52], in a level IV study analyzed the repair integrity and clinical outcome following arthroscopic double-row rotator cuff repair and reported that this technique can lead to improved repair integrity compared with open or mini-open repair methods. They also concluded that the retear rate for shoulders with large or massive tears remains higher than that for smaller tears, and shoulders with large defects demonstrated significantly inferior functional outcomes, whereas a small defect remaining after surgery did not have an adverse effect on the postoperative function. According to their results, the authors believe that the function of the rotator cuff in maintaining the humeral head centered against the glenoid fossa is well preserved in shoulders even with a small defect and therefore we probably do not have to be overly concerned about postoperative small rotator cuff defects detected by magnetic resonance imaging or ultrasonography.

In a recent systematic review and meta-analysis, McElvany *et al*. [2], found that the clinical outcomes were generally improved despite a mean retear rate of 26.7%. Finally, a certain number of studies suggest [38, 53-55] that there are no significant differences in the shoulder scores between these two groups, particularly in terms of patient satisfaction both in short and long term [56] follow up.

## DISCUSSION

Structural failure or re-tear after rotator cuff repair is a well described and frequently encountered complication [57, 58]. Therefore, one of the most challenging issues in rotator cuff surgery is to restore anatomy, solidly fix tendon to bone and substantially increase the rate of healing. Postoperatively, the most commonly used imaging modalities are ultrasonography, magnetic resonance imaging (MRI) or CT arthrography [20, 59]. MRI scan is considered the primary investigative tool for evaluation cuff integrity with higher sensitivity and specificity compared to other imaging studies [11-13, 31, 48].

There seems to be lack of robust evidence to support our hypothesis that re-tears after rotator cuff repair lead to poorer clinical outcome and restriction in daily activities [60, 61]. The results show that this failure does not necessarily lead to poor clinical outcome at least in the short- or mid-term follow up. However, there seems to be a trend towards clinical deterioration in the long-term period after tendon tear recurrence, as was implied from a few clinical studies [46-49]. Also it is obvious that the larger the postoperative defect is, the poorer the clinical outcome ensues. Finally, even in studies with no statistically significant difference in function between the healed and the failed repair groups, muscle strength in external rotation, abduction and forward flexion is notably higher in the healed group.

Regarding the factors predicting the risk of structural failure after RC repair, there are several patient-related factors that negatively affect tendon to bone healing: older patient age, poor muscle quality with extensive fatty infiltration, greater degree of muscle-tendon unit retraction, larger anteroposterior and mediolateral length tear and overall size, and various systemic comorbidities, such as smoking, diabetes, osteoporosis and hypercholesterolemia [14]. There are also surgeon-related factors that are recognized to potentially affect the rate of healing. More recent studies suggest that double-row suture-bridge transosseous-equivalent techniques are superior to previous traditional double-row non-linking techniques or single-row techniques as they seem to offer stronger initial mechanical fixation of tendon to bone and better recreate the anatomic footprint onto the greater tuberosity [32, 34]. In fact, there is little evidence to support the presence of significant functional differences between the 2 techniques, except possibly for patients with large or massive rotator cuff tears (>3 cm). Well-designed large prospective randomized studies with homogenous techniques and study populations are therefore needed in the future to definitively settle this debate. Furthermore, slower rehabilitation with prolonged immobilization seems to improve healing rate and functional outcome in patients with full-thickness tears.

## CONCLUSION

Our study shows that there is still lack of high-level prospective studies that directly correlate the clinical outcome with the restoration of rotator cuff anatomy. However, there is a good number of studies to support that anatomic restoration of the torn rotator cuff by implementing the newer arthroscopic techniques can lead to higher healing rates, greater muscle strength and better overall function and patient satisfaction, particularly in younger patients with higher demands. On the other hand, there are certain studies which could not find any significant difference in clinical outcome between patients with healed cuff and those with structural failure.

## Figures and Tables

**Table 1:** Comparison of re-tear rates for different rotator cuff repair techniques.

**Author**	**Year**	**Level of evidence**	**Sample**	**Follow-up**	**Technique**	**Outcome**	**Re-tear rate**	**Comparison**
**Shen *et al*. [30]**	2014	Systematic review, meta-analysis	428 patients/6 studies	>6 months (different for each study)	Single-row vs double-row	ASES, Constant, UCLA	Risk ratio for double-row 1,71(95% CI) RR for single-row 2,16 (95% CI)	1) Functional scores: no difference between single and double row technique 2) Double-row technique decreased the incidence of re-tears (especially partial-thickness) compared to single-row 3) No difference to clinical outcome between the 2 techniques
**Kim *et al*. [33]**	2014	Cohort study level III	65 patients with retear after full-thickness rotator cuff tear repair	>6months	Single-row technique (SRT) Suture-bridge technique (SBT) Knotless suture-bridge technique (K-SBT)	MRI at least 6months postoperative Type 1: unhealed tendons Type 2: medially ruptured tendons with a healed footprint Type 3: unable to classify	-	1) 21 patients SRT 22 patients SBT 22 patients K-SBT 2) Type 1: 71,4% in SRT 40,9% in SBT 54,5% in K-SBT Type 2: 23,8% in SRT 59% in SBT 40,9% in K-SBT 3) No significant difference between 3 groups (p=0,195) 4) Significant difference between SRT and SBT groups alone (p=0,049) 5) No significant differences for either type 1 (p=0,121) or type 2 retears (p=0,064) among 3 groups 6) No significant differences in type 1 (P=0,281) or type 2 full-thickness re-tears (P-0,117) among 3 groups 7) In pairs group comparison, significant difference in type 2 full-thickness re-tears between SRT and SBT groups alone (P=0,037) 8) Conclusion: SBT has different retear pattern than SRT, K-SBT retear pattern is no different from that of SRT
**Nho *et al*. [7]**	2009	Level III, systematic review of levels I to III	All studies from 1966 to 2008 which compare SRT to DRT Excluded the studies that lacked comparison group (case series)-only 5 studies remained	-	-	-	-	1) No clinically significant differences between SRT and DRT 2) Some studies report that DRT may improve tendon healing
**Mascarenhas *et al*. [60]**	2014	Level II, systematic review of level I and II studies	8 meta-analyses (4 level I and 4 level I and II studies)	-	SR, DR	Oxman-Guyatt scores	-	1) 6 meta-analyses no difference between SR and DR for patient outcomes 2) 2 favored DR vs SR for tears >3cm 3) 2 no structural healing differences between DR and SR 4) 3 DR superior to SR for tears>3cm 5) 2 DR superior to SR for all tears 6) 4 had Oxman-Guyatt scores<3 = major flaws

ASES: American Shoulder and Elbow Surgeons, DRT: double-row technique, K-SBT: knotless suture-bridge technique, RR: risk ratio, SBT: suture-bridge technique, SRT: single-row technique, UCLA: University of California at Los Angeles

**Table 2:** Clinical studies showing better results in patients with healed rotator cuff repair compared to structurally failed repair.

**Author**	**Year**	**Level of evidence**	**Sample**	**Follow-up**	**Technique**	**Outcomes**	**Conclusion**
**Vastamäki *et al*. [46]**	2013	Level IV, therapeutic study	67 patients Mean age 52 years	Minimum 16 years Range 16-25 years	Open repair	MR arthrography	1) Re-tear rate 94%, Mean size of re-rupture (3,5x3,6cm) 2) 6% partial tear of supraspinatus 3) Fatty infiltration in supraspinatus and infraspinatus tendons 4) Active external rotation and forward flexion, strength of flexion, abduction and external rotation were better in patients with intact rotator cuff or small re-tear <4cm 5) Cuff integrity correlated with functional results several years postoperatively
**Park *et al*. [47]**	2013	Retrospective level IV study	36 patients with massive tear	37,6 +/- 8,9 months	Arthroscopic suture bridge repair	US (4.5, 12 and 24 months postoperative) ASES, ROM, Constant and muscle power	1) 25% recurrent tear, 75% complete healing 2) All functional scores improved, but the re-tear group (especially with large size) had poorer outcome than healed group (ASES P=0.005, Constant P=0.175) 3) Fatty degeneration of supraspinatus preoperatively associated with high re-tear rate
**Zumstein *et al*. [45]**	2008	-	27 patients with massive tear	9.9 years	Open repair	Constant score Radiographs and MRI	1) Re-tear rate 57% at 9.9 years and 37% at 3.1 years 2) Patients with an intact repair had better absolute and relative Constant score and abduction strength than those with failed reconstruction 3) Re-tear size increased from the initial 4) Supraspinatus and infraspinatus muscle fatty infiltration increased 5) Acromion index higher in re-tear group than intact group
**Yoo *et al*. [3]**	2013	Level III cohort study	81 patients	29.7 months	-	SF-36 scores, UCLA, ASES	1) 56/81 in healed group 25/81 in re-tear group 2) Clinical scores were significantly improved in both groups but significantly higher in the healed group
**Kim *et al*. [49]**	2014	cohort	180 patients	At least 1 year	-	US ASES, SST	1) Clinical scores significantly poorer in the re-tear group (p<0,05) 2) Patients with a re-tear, non-anatomic factors including younger age, lower educational level and heavy workers were associated with poorer outcomes
**Lafosse *et al*. [50]**	2007	-	105 patients	-	Arthroscopic double-row repair	MRI arthrography, CT Constant score, muscle strength, ROM	1) Superior clinical outcome in patients with healed repairs but not statistically significant 2) Pain relief in healed group (statistically significant p=0,014)

ASES: American Shoulder and Elbow Surgeons, CT: computed tomography, MRI: magnetic resonance imaging, ROM: range of motion, SST: simple shoulder test, UCLA: University of California at Los Angeles, US: ultrasound

**Table 3:** Clinical studies showing no difference in clinical outcome between patients with healed and structurally failed rotator cuff repairs.

**Author**	**Year**	**Level of evidence**	**Sample**	**Follow-up**	**Technique**	**Outcomes**	**Conclusion**
**Jost *et al*. [44]**	2000	Prospective	20 patients (mean age 59 years)	-	Open repair	MRI evaluation	1) 16/20 patients smaller re- rupture 2) Fatty degeneration of SS and IS, atrophy of SS and GH osteoarthritis progressed significantly 3) Clinical outcome significantly correlated with postoperative tear, stage of postoperative fatty degeneration of IS and SSC, postoperative acromiohumeral distance, postoperative GH osteoarthritis (p<0,05) Finally: significantly decreased pain (p=0,0026) and improved function (p=0,0005) and strength (p=0,0137) despite failure of repair
**Voigt *et al*. [35]**	2010	Level IV	51 patients	12 months	Arthroscopic suture bridge repair of supraspinatus	MRI SST and Constant score	1) Re-tear rate 28,9% with no significant difference in clinical outcome between intact R.C. and re-tear group, but structural failure is not compatible with clinical failure 2) Age>60 negatively influenced tendon healing
**Kim *et al*. [37]**	2012	Retrospective	77 patients	-	Arthroscopic suture bridge repair of full thickness cuff tears	MRI U/S UCLA, ASES, Constant-Murley scores	1) Postoperative clinical outcomes improved in all patients without difference between healed R.C. and structural failure (p=0,438, p=0,625 and p-0,898 for UCLA, ASES and Constant score
**Rhee *et al*. [51]**	2014	Level III case-control study	238 patients (two groups>70 years old and <70 years old)	Short mean follow-up (at least 6 months)	-	MRI	1) Both groups significant improvement in clinical outcomes with no significant difference between (p=0,161) 2) Retear rate 39,8% <70, 51,1% >70 3) Retear rate increased significantly depending on intraoperative size but not on age 4) No comparison of the functional outcome between re-tear and intact R.C. groups
**McElvany*et al*. [2]**	2015	Systematic review and meta-analysis	-	At least 6 months	All techniques	Radiological	1) Mean re-tear rate 26,6%2) Clinical outcomes were improved both in re-tear and in intact R.C. group 3) Re-tear rate associated with greater degree of fatty infiltration, larger tear size, advanced age and double-row repairs
**Lubiatowski *et al*. [53]**	2012	Retrospective study of 111 cases	111 cases	At least 6 months	All techniques	UCLA, ASES and SST scores Radiological	1) No significant difference in shoulder scores and patients’ satisfaction depending on quality of healing 2) Incomplete R.C. healing in 26% of cases 3) R.C. integrity after open or arthroscopic repair did not seem to affect clinical scores although recurrent tears may result in lower muscle strength, endurance and active motion
**Russell *et al*. [61]**	2014	Systematic review and meta-analysis of Level I and Level II studies	14 studies (861 patients)	At least 1 year	All techniques	UCLA, ASES, Constant score	1) Not clinically important improvement regardless of the structural integrity of the repair 2) Patients with intact repairs significantly greater strength in forward elevation and external rotation to those with retears
**Choi *et al*. [54]**	2012	Comparative study	41 arthroscopic rotator cuff repair	28 months (average)	Double-pulley suture bridge repair	ASES, Constant score, UCLA	1) Retear rate 19,5% 2) 75% within 6 months after operation and 25% >1year 3) Functional and clinical improvement independent of tear size and R.C. integrity
**Kim *et al*. [49]**	2014	Level IV retrospective study	24 patients with full thickness rotator cuff tear	-	-	MRI and ultrasound scan ASES, VAS, UCLA, Constant-Murley, ROM	1) Retear rate of 47,8% (smaller size than the initial) 2) No significant difference in clinical results between intact and retear group
**Sugaya *et al*. [52]**	2007	Level IV study	106 patients	At least 6 months	Arthroscopic double-row rotator cuff repair	MRI and ultrasonography	1) Arthroscopic double-row rotator cuff repair improved integrity compared with open and mini-open repair 2) Re-tear rates depend on initial tear size 3) Functional improvement depends on initial tear size 4) Function of R.C. remains even when small R.C. defects are recognized postoperatively by MRI
**Paxton *et al*. [56]**	2013	-	-	10 years	-	ASES, SST and Constant scores Ultrasound	Clinical improvement to those patients despite re-tear Conclusion: no structural healing is critical for massive tears due to the long-term satisfactory results at least in older patients
**Moraiti *et al*. [55]**	2015	Multicenter, prospective, comparative study of 40 patients <50 years and 40 >70 years Level IV therapeutic case series	80 patients	1 year	Arthroscopic repair	MRI and ultrasound Constant and modified Constant scores, patients’ satisfaction	1) Healing rate lower in the older age group which was characterized by greater retraction in frontal plane and greater fatty infiltration 2) Functional outcome and satisfaction equal to both groups

ASES: American Shoulder and Elbow Surgeons, GH: glenohumeral, IS: infraspinatus, MRI: magnetic resonance imaging, R.C.: rotator cuff, SS: supraspinatus, SSC: subscapularis, SST: Simple Shoulder Test, UCLA: University of California at Los Angeles, VAS: visual analog scale

## LIST OF ABBREVIATIONS

ASES: = American shoulder and elbow surgeons
CT: = Computed tomography
MRI: = Magnetic resonance imaging
RC: = Rotator cuff
ROM: = Range of motion
SST: = Simple Shoulder Test
UCLA: = University of california at los Angeles
VAS: = Visual analogue scale
